# Reactivity of Cyclanols Towards Quinaldinium Fluorochromate Oxidation

**DOI:** 10.1007/s10953-013-0070-2

**Published:** 2013-09-29

**Authors:** K. G. Sekar, R. V. Sakthivel

**Affiliations:** 1 Department of Chemistry, National College, Tiruchirappalli, 620001 Tamil Nadu India; 2 Department of Chemistry, Arignar Anna Government Arts College, Namakkal, 637002 Tamil Nadu India

**Keywords:** Oxidation, Cyclanols, Quinaldinium fluorochromate, Kinetics

## Abstract

The kinetics of oxidation of cyclanols, viz., cyclohexanol, cyclopentanol, cycloheptanol and cyclooctanol by quinaldinium fluorochromate has been studied in aqueous acid medium at 313 K (±0.1 K). The cyclanols were converted to the corresponding cyclic ketones. The order of reaction was found to be one with respect to oxidant and fractional with respect to the substrate and hydrogen ion concentrations. Increase in the percentage of acetic acid increases the rate of reaction. The reaction mixture shows the absence of any free radicals in the reaction, which has ruled out the possibility of a one-electron transfer during the addition of acrylonitrile. The reaction has been studied at four different temperatures and the activation parameters were calculated. From the observed kinetic results a suitable mechanism was proposed. The relative reactivity order was found to be cyclohexanol < cyclopentanol < cycloheptanol < cyclooctanol. This was explained on the basis of I-strain theory.

## Introduction

Quinaldinium fluorochromate (QnFC) [1], a Cr(VI) compound, has been reported to be a neutral and mild oxidant for selective oxidation reactions. The kinetics of oxidation of some organic substrates by quinaldinium fluorochromate have already been reported. The kinetics of oxidation of cyclanols with various oxidants show reactivities that varies with the type of oxidant [2–11]. The differences in the reactivities have been explained by the I-strain theory.

The probable structure of quinaldinium fluorochromate (QnFC) is the following:  The present study on the oxidation of cyclanols by quinaldinium fluorochromate is to ascertain the nature and the order of reactivity of these compounds under the given kinetic conditions.

## Experimental

### Materials

All the cyclanols, cyclohexanol, cyclopentanol, cycloheptanol and cyclooctanol were purchased from Sigma Aldrich Company and were of AnalaR grade (99.9 %). This reported purity was checked from physical constants (boiling point) of the cyclanols. Boiling points of the cyclanols, viz., cyclohexanol = 160 °C (lit. 158–161 °C), cyclopentanol = 140 °C (lit. 141 °C), cycloheptanol = 185 °C (lit. 186 °C), and cyclooctanol = 107 °C (lit. 105–108 °C). Quinaldinium fluorochromate was prepared by a reported method [1] and its purity was checked by estimating Cr(VI) iodometrically (yield 85 %). The structures of the products were confirmed by elemental analysis and their IR (in KBr) spectra; the IR frequencies of the fluorochromate group occur at *v* = 948, 870 and 617 cm^−1^ in QnFC. All other chemicals used were AnalaR grade. Acetic acid was refluxed over chromic oxide and acetic anhydride and then fractionally distilled. The fraction boiling at 116–118 °C was collected and kept in a brown bottle. Doubly distilled water was used throughout the measurements. The reaction mixture was homogeneous throughout the course of the reaction.

### Kinetic Measurements

The reactions were performed in aqueous of acetic acid, under pseudo-first order conditions, by maintaining a large excess of substrate over quinaldinium fluorochromate. The kinetic measurements were carried out spectrophotometically in a thermostated cell compartment of a spectrophotometer (Perkin Elmer, Lambda 35) at 470 nm. This wavelength of the maximum absorption due to quinaldinium fluorochromate has been observed and absorption due to other reaction species is negligible [12]. The oxidation reaction was studied only in the concentration range of quinaldinium fluorochromate where the Beer’s law is obeyed. The reactions were carried out at constant temperature 313 K (±0.1 K) followed up to 70 % completion. The rate constants were evaluated from the linear plot of log_10_ (absorbance) versus time by the least-squares method and were reproducible to within ±3 %.

### Stoichiometry and Product Analysis

Reaction mixtures containing an excess of the oxidant over cyclanols were kept at room temperature in the presence of perchloric acid for two hours. Estimation of the unreacted oxidant proved that one mole of oxidant consumes one mole of substrate. The same experimental conditions were used for kinetic determinations; a solution of the reaction mixture was kept under nitrogen for 24 h. The solution was then extracted with ether, the organic layer washed with water, dried over anhydrous sodium sulfate and then concentrated. The product cyclohexanone was identified by a spot test [13]. It was dissolved in DMF and tlc analysis was done with cyclohexanone and a standard sample of cyclohexanone as reference. Only one spot corresponding, to cyclohexanone, was obtained. The product was further confirmed by IR spectral data, which show a peak at 1710 cm^−1^ that corresponds to the carbonyl group of cyclohexanone.

## Results and Discussion

The kinetics of oxidation of cyclohexanol (CHOL) by quinaldinium fluorochromate was investigated at several initial concentrations of the reactants. The oxidation of cyclohexanol by quinaldinium fluorochromate proceeds smoothly at 313 K in aqueous acetic acid medium and the observed results are discussed below.

### Effect of Varying the Oxidant Concentration

The reaction was found to be first order with respect to the oxidant as evidenced by good linearity in the plot of log absorbance versus time (*r* = 0.996) (Table 1). The pseudo-first order rate constants were found to be independent of the initial concentration of quinaldinium fluorochromate.

**Table 1:** Rate data on the oxidation of cyclohexanol by quinaldinium fluorochromate at 313 K. Solvent: AcOH + H_2_O = 60 : 40 (%, v/v)

[CHOL] × 10^2^(mol·dm^−3^)	[QnFC] × 10^4^ (mol·dm^−3^)	[H^+^] × 10^2^ (mol·dm^−3^)	*k*_1_ × 10^4^ (s^−1^)
1.5	10.5	3.0	8.00
3.0	10.5	3.0	12.17
4.5	10.5	3.0	14.18
6.0	10.5	3.0	16.20
7.5	10.5	3.0	17.32
3.0	7.0	3.0	12.22
3.0	10.5	3.0	12.17
3.0	14.0	3.0	12.26
3.0	17.5	3.0	12.23
3.0	21.0	3.0	12.27
3.0	10.5	1.5	10.11
3.0	10.5	4.5	14.31
3.0	10.5	6.0	14.95
3.0	10.5	7.5	15.95

### Effect of Varying the Substrate Concentration

**Fig. 1:**
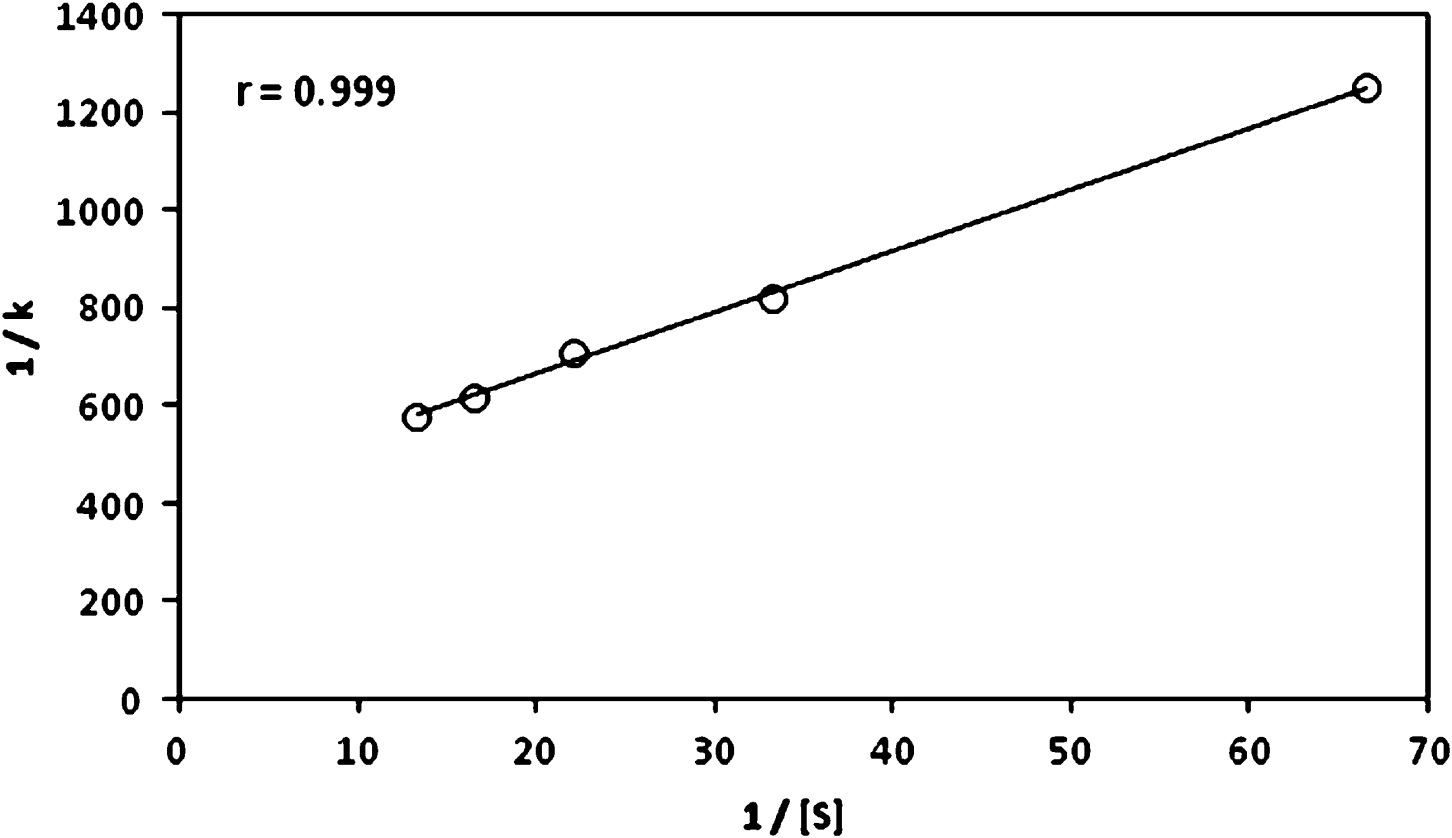
Plot of 1/*k* versus 1/[s]

The order with respect to substrate was found to be fractional as evidenced by the linear plot of log_10_ *k* versus log_10_ [substrate] with a slope of 0.48. It was further supported by the fact that the plot of 1/*k* versus 1/[s] gave a straight line (Fig. 1) with a definite intercept, indicating a Michaelis–Menten type of kinetics for the reaction (Table 1).

### Effect of Perchloric Acid Concentration

**Fig. 2:**
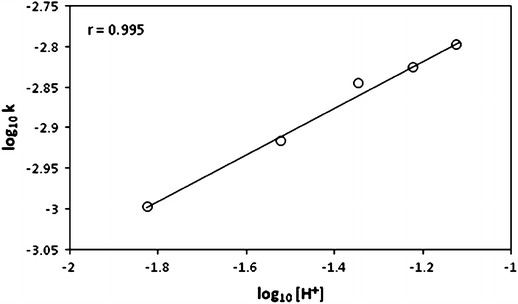
Plot of log_10_ *k* versus log_10_ [H^+^]

At constant concentrations of the reactants and at constant ionic strength, the rate constants increased with increase in the concentration of perchloric acid as seen in Table 1. This shows the participation of perchloric acid in the rate determining step and the order has been found to be 0.29 from the plot of log_10_ *k* versus log_10_ [H^+^] (Fig. 2).

### Effect of Ionic strength and Dielectric Constant of the Medium

**Fig. 3:**
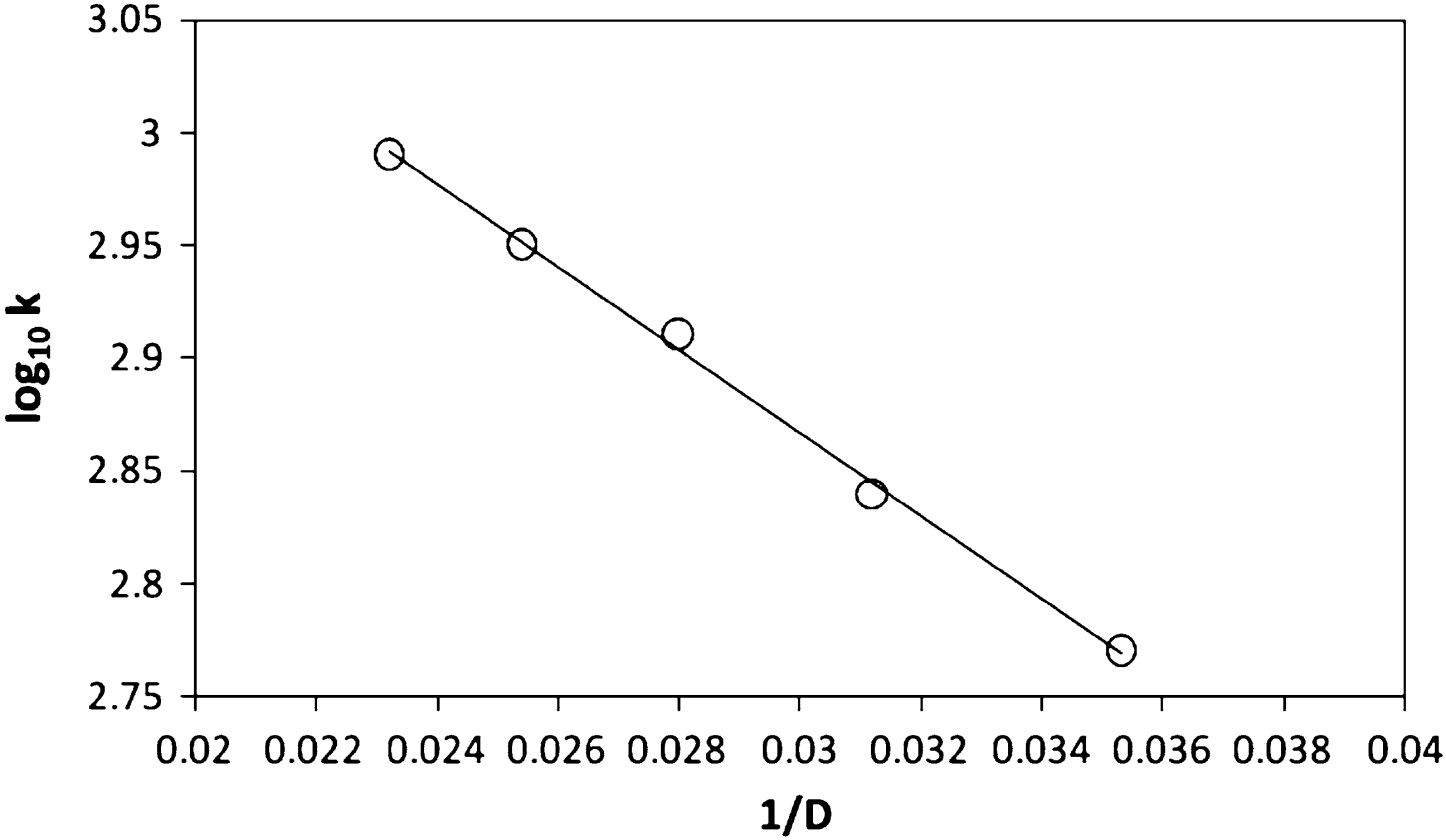
Plot of log_10_ *k* versus 1/*D*

An increase in the ionic strength of the medium from adding sodium perchlorate had no effect on the reaction rate indicating the involvement of a neutral molecule in the rate determining step. The rates were found to increase with increase in the percentage of acetic acid. A plot of log_10_ *k* versus *D* ^−1^ is linear with a positive slope (Fig. 3). This suggests an interaction between a positive ion and neutral molecule. It also confirms the involvement of protonated Cr(VI) species in the rate determining step (Table 2).

**Table 2:** Dependence of rate constant on ionic strength, solvent composition, and [MnSO_4_] at 313 K: [CHOL] = 3.0 × 10^−2^ mol·dm^−3^, [QnFC] = 10.5 × 10^−4^ mol·dm^−3^, and [H^+^] = 3.0 × 10^−1^ mol·dm^−3^

[NaClO_4_] × 10^2^ (mol·dm^−3^)	AcOH:H_2_O (%, v/v)	[MnSO_4_] × 10^2^ (mol·dm^−3^)	*k*_1_ × 10^4^ (s^−1^)
0.00	60–40	–	12.17
2.50	60–40	–	12.16
5.00	60–40	–	12.24
7.50	60–40	–	12.34
10.00	60–40	–	12.12
–	50–50	–	10.17
–	55–45	–	11.23
–	65–35	–	14.45
–	70–30	–	16.98
–	60–40	0.0	12.17
–	60–40	5.01	11.05
–	60–40	10.03	10.80
–	60–40	15.04	10.54
–	60–40	20.06	10.26

The reaction mixture when allowed to stand with acrylonitrile does not induce polymerization, suggesting the absence of a free radical mechanism. On the other hand, the addition of Mn^2+^ ion retards the rate of the reaction. This suggests a two-electron transfer in the rate determining step [14] (Table 2).

### Effect of Temperature

**Fig. 4:**
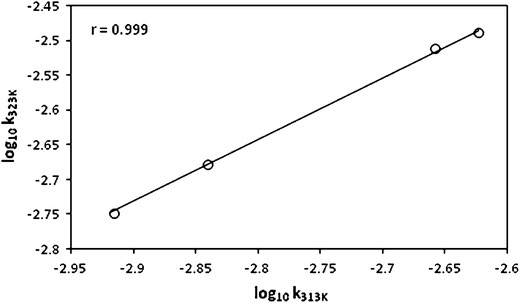
Plot of log_10_ *k*_323 K_ versus log_10_ *k*_313 K_

The oxidation reaction was conducted at four different temperatures viz., 303, 313, 323 and 333 K and the measured rate constant values are given in Table 3. An increase in temperature resulted in an increase in the rate of reaction. The thermodynamic parameters were calculated by using Eyring’s [15] plot of ln *k*_obs_/*T* versus 1/*T*. The negative values of the entropy of activation (Δ*S* ^#^) suggested extensive solvation of the transition state compared to the reactants. The values (Table 3) of the Gibbs energies of activation (Δ*G* ^#^) were fairly constant indicating that a similar mechanism operated for the oxidation of all the cyclanols studied. As (Δ*H* ^#^) and (Δ*S* ^#^) do not vary linearly, no isokinetic relationship is observed. This indicates the absence of an enthalpy–entropy compensation effect [16]. The linear Exner’s plot [17] (Fig. 4) favors a similar mechanism for all of the cyclanols.

**Table 3:** Rate constants and thermodynamic parameters for the oxidation of cyclanols by QnFC: [cyclanols] = 3.0 × 10^−2^ mol·dm^−3^, [QnFC] = 10.5 × 10^−4^ mol·dm^−3^, [H^+^] = 3.0 × 10^−1^ mol·dm^−3^, and AcOH : H_2_O = 60 : 40 (%, v/v)

Cyclanols	*k*_1_ × 10^4^ (s^−1^)				Δ*H* ^#^ (kJ·mol^−1^)	−Δ*S* ^#^ (J·K^−1^·mol^−−1^)	Δ*G* ^#^ (kJ·mol^−1^) at 313 K	*E*_a_ (kJ·mol^−1^) at 313 K	*r*
	303 K	313 K	323 K	333 K					
Cyclohexanol	9.00	12.17	17.84	25.32	26.56	186.94	94.26	29.16	0.996
Cyclopentanol	10.57	14.46	20.93	29.13	25.97	187.48	93.80	28.57	0.997
Cycloheptanol	16.8	21.98	30.76	42.23	24.46	188.92	92.72	27.06	0.998
Cyclooctanol	17.07	23.81	32.41	45.24	24.48	188.29	92.50	27.08	0.999

Error limits: Δ*H* ^#^ 25.97 ± 2 kJ·mol^−1^, Δ*S* ^#^ −187.48 ± 4 J·K^−1^·mol^−1^, and Δ*G* ^#^ 93.80 ± 2 kJ·mol^−1^ at 313 K

Considering all the above facts and the thermodynamic parameters, the following mechanism has been proposed.

### Rate Law

The above mehanism was substantiated by the following rate law:

$$ {\text{rate }} = - d\left[ {\text{QnFC}} \right] \, /dt = k_{ 3} \left[ {\text{complex}} \right] $$

By applying steady state approximation for the complex formed in Step 2.

$$ {\text{rate}} = \frac{{k_{3} K_{2} \left[ {\text{s}} \right]\left[ {C_{1} } \right]}}{{1 + K_{2} \left[ {C_{1} } \right]}} \; = \;\frac{{k_{3} K_{2} K_{1} \left[ {\text{QnFC}} \right]\left[ {{\text{H}}^{ + } } \right]}}{{\left( {1 + K_{2} \left[ {\text{s}} \right]} \right)\left( {1 + K_{1} \left[ {{\text{H}}^{ + } } \right]} \right)}}\; = \;\frac{{k_{3} K_{2} K_{1} \left[ {\text{s}} \right]\left[ {\text{QnFC}} \right]\left[ {{\text{H}}^{ + } } \right]}}{{1\; + \;K_{2} \left[ {\text{s}} \right]\left[ {{\text{H}}^{ + } } \right]\; + \;K_{1} \left[ {{\text{H}}^{ + } } \right]}} $$

Since, *K*_1_ *K*_2_ [s] [H^+^] ≪ 1:

$$ {\text{rate }} = \frac{{k_{ 3} K_{ 2} K_{ 1} \left[ {\text{s}} \right]\left[ {{\text{H}}^{ + } } \right]}}{{ 1+ K_{ 2} \left[ {\text{s}} \right] \, + {\text{ K}}_{ 1} \left[ {{\text{H}}^{ + } } \right]}} $$

This rate law explains all the observed experimental facts.

### Structure and Reactivity

The rates of the reaction for all the cyclanols have been rationalized by the difference in strain energy between the ground state and transition state of the molecule in the process considered [15]. In the present study, the order of reactivity of cyclanols by quinaldinium fluorochromate is found to be cyclohexanol < cyclopentanol < cycloheptanol < cyclooctanol.

In the cyclohexane ring system, there is a non-bonded interaction between the flagpole and bowsprit positions. The reaction occurs in its flexible boat form that has bond opposition strain though not an angle strain involving four pairs of hydrogen at the side of the boat.

In the cyclopentane ring system, the angle strain may not be appreciable but there will be a strain due to the eclipsing interaction with the adjacent hydrogen atoms. The larger rate of reduction of cyclohexanone with sodium borohydride compared to that of cyclopentanone is ascribed to the increased torsional strain caused by the conversion of sp^2^ to sp^3^ hybridization, since in six membered ring systems the conversion of sp^2^ to sp^3^ is easier because of the small bond opposition. The higher rates of cycloheptanol and cyclooctanol oxidation are due to the largest I-strain involved during the sp^3^ to sp^2^ change [8].
